# Real-Time Compressive Sensing MRI Reconstruction Using GPU Computing and Split Bregman Methods

**DOI:** 10.1155/2012/864827

**Published:** 2012-02-01

**Authors:** David S. Smith, John C. Gore, Thomas E. Yankeelov, E. Brian Welch

**Affiliations:** ^1^Institute of Imaging Science, Vanderbilt University, Nashville, TN 37232, USA; ^2^Department of Radiology and Radiological Sciences, Vanderbilt University, Nashville, TN 37232, USA; ^3^Department of Biomedical Engineering, Vanderbilt University, Nashville, TN 37232, USA; ^4^Department of Molecular Physiology and Biophysics, Vanderbilt University, Nashville, TN 37232, USA; ^5^Department of Physics and Astronomy, Vanderbilt University, Nashville, TN 37232, USA; ^6^Department of Cancer Biology, Vanderbilt University, Nashville, TN 37232, USA

## Abstract

Compressive sensing (CS) has been shown to enable dramatic acceleration of MRI acquisition in some applications. Being an iterative reconstruction technique, CS MRI reconstructions can be more time-consuming than traditional inverse Fourier reconstruction. We have accelerated our CS MRI reconstruction by factors of up to 27 by using a split Bregman solver combined with a graphics processing unit (GPU) computing platform. The increases in speed we find are similar to those we measure for matrix multiplication on this platform, suggesting that the split Bregman methods parallelize efficiently. We demonstrate that the combination of the rapid convergence of the split Bregman algorithm and the massively parallel strategy of GPU computing can enable real-time CS reconstruction of even acquisition data matrices of dimension 4096^2^ or more, depending on available GPU VRAM. Reconstruction of two-dimensional data matrices of dimension 1024^2^ and smaller took ~0.3 s or less, showing that this platform also provides very fast iterative reconstruction for small-to-moderate size images.

## 1. Introduction

Magnetic resonance imaging (MRI) is an important application of compressive sensing (CS) [1–4]. CS in MRI [5] has the potential to significantly improve both the speed of acquisition and quality of MR images, but requires an iterative reconstruction that is more computationally intensive than traditional inverse Fourier reconstruction. Compressive sensing accelerates MR acquisitions by reducing the amount of data that must be acquired. Reconstruction of this partial data set is then accomplished by iteratively constraining the resulting image to be sparse in some domain while enforcing consistency of the measured subset of Fourier data.

 One practical barrier to the routine adoption of CS MRI is the delay between acquisition and reconstruction of images. Compressed sensing solvers work almost entirely with vector and image arithmetic, making them an excellent candidate for acceleration through using graphics processing units (GPUs) for parallelization. Here we illustrate how GPUs can be used to achieve significant increases in speed of CS reconstructions of large MRI data sets.

GPU computing means using the GPU to perform general purpose scientific and engineering computation. High-end video cards can contain hundreds of separate floating-point units, allowing for massively parallel computations at a fraction of the cost of CPU-based supercomputers, as measured on a per gigaFLOP basis (one gigaFLOP is equivalent to one billion floating point operations per second). GPU computing works best in single instruction, multiple data (SIMD) situations, such as the solution of large systems of linear equations. The power of GPU computing is already being realized in several advanced medical image reconstruction applications [6–9].

## 2. Materials and Methods

### 2.1. Hardware

The reconstruction platform tested was a high-performance GPU server designed to serve an MRI scanner as a dedicated reconstructor. This system contained a six-core, 2.67 GHz Xeon X5650 with 12 GB of 1333 MHz DDR3 RAM, 32 KB of L1 cache, 256 KB of L2 cache, and 12 MB of L3 cache. An NVIDIA Tesla C2050 was used for this experiment. The Tesla C2050 contains 448 CUDA cores arrayed as 14 Streaming Multiprocessors (SMs) with 32 cores per SM. It has 3 GB of VRAM and 64 KB of memory per SM. All SMs share 768 KB of L2 cache. The Tesla C2050 has a theoretical maximum double-precision performance of 515 GFLOPs.

### 2.2. Reconstruction Problem

The optimization problem we are choosing to explore is the reconstruction of partial MRI data sets using CS. MRI is proving to be a fertile application for CS because many MR images can be highly compressed by transform coding with little loss of information. Conventional MRI data is acquired according to the Fourier sampling pattern required to satisfy the Nyquist criterion while producing an image of a given field of view and spatial resolution. To sample the Fourier domain “compressively,” only a random subset of the full Nyquist Fourier sampling scheme is acquired. Reconstruction of the conventional fully sampled MRI data requires simply an inverse Fourier transform,

(1) $$u=F^{-1}b,$$

but the inverse problem becomes underdetermined when part of the Fourier data is omitted, so approximate methods must be used.

 One highly successful method in particular has been to formulate the CS MRI reconstruction as a sparse recovery problem, in which an image is found that is consistent with the acquired Fourier data while having the sparsest representation in a chosen basis (e.g., gradient and wavelet). The typical formulation of the reconstruction of a complex image *u* from a partial Fourier data set *b* is then

(2) $$u=\underset{x}{\arg\;\min}{||x||}_{1}+\lambda {||Ax-b||}_{2}^{2},$$

where *A* is a measurement operator that transforms the sparse representation *x* to the image domain then performs the subsampled Fourier measurement, and the *l*_1_ and *l*_2_ norms are, respectively,

(3) $$\begin{array}{c} {||x||}_{1}=\underset{i}{\sum }\left|x_{i}\right|,\\ {||x||}_{2}^{2}=\underset{i}{\sum }{\overset{̅}{x}}_{i}x_{i}, \end{array}$$

where the bar denotes complex conjugation. With the addition of the *l*_1_ norm, the problem is more difficult to solve, and iterative techniques such as interior point methods [10, 11], iterative soft thresholding [12, 13], and gradient projection [14–16] are typically employed.

### 2.3. Software

The open-source split Bregman code of Goldstein and Osher [16] was chosen as the starting point for the GPU-based CS solver. The solver was originally written in Matlab. This solver was chosen for its rapid convergence and lack of array reduction steps, which hinders parallelization. We modified the original code to work with Jacket 1.8.0 (AccelerEyes, Atlanta, GA) and Matlab R2010b (Mathworks, Natick, MA). CUDA Toolkit 4.0 and CUDA developer driver 270.41.19 were used for all computations.

Algorithm 1 briefly outlines the procedure for running the split Bregman reconstruction on the GPU. Note that the split Bregman algorithm runs for a fixed number of iterations, so there is no variation in run time due to different descent trajectories as with a tolerance-based stopping criterion. Furthermore, the choice of image reconstructed has no bearing on the results, since the fixed number of iterations ensure that the same number of operations are performed on any input data set.

 At the beginning of the reconstruction, the Fourier data in main memory must be transferred to the GPU with Jacket's *gsingle* and *gdouble* Matlab commands. Next, temporary storage is allocated on the GPU using Jacket's *gzero* and *gones* commands. All subsequent arithmetic operations, including the Fourier transform, are carried out on the GPU using function overloading. Function overloading simplifies code syntax by enabling a single function to encapsulate different functionality for different types of arguments. The specific behavior is typically chosen by the compiler or at run time. In our case, Matlab automatically calls the Jacket library if an operation is requested on a matrix that lies in GPU memory, while identical operations on a matrix in main memory are carried out with Matlab's built-in functions. After the last loop iteration on the GPU, the solution is transferred back to main memory with overloaded versions of Matlab's *double* and *single* commands; all temporary storage is automatically freed.

### 2.4. Experiments

Two numerical experiments were performed. The first was a pure matrix multiplication, designed to measure practical peak floating-point performance of the CPU and GPU as realized by Jacket 1.8.0. To remove dependencies on the multithreading performance of Matlab R2010b and provide easier comparison of the CS reconstructions, the CPU experiment was run both with and without multithreading enabled.

**Figure 1:**
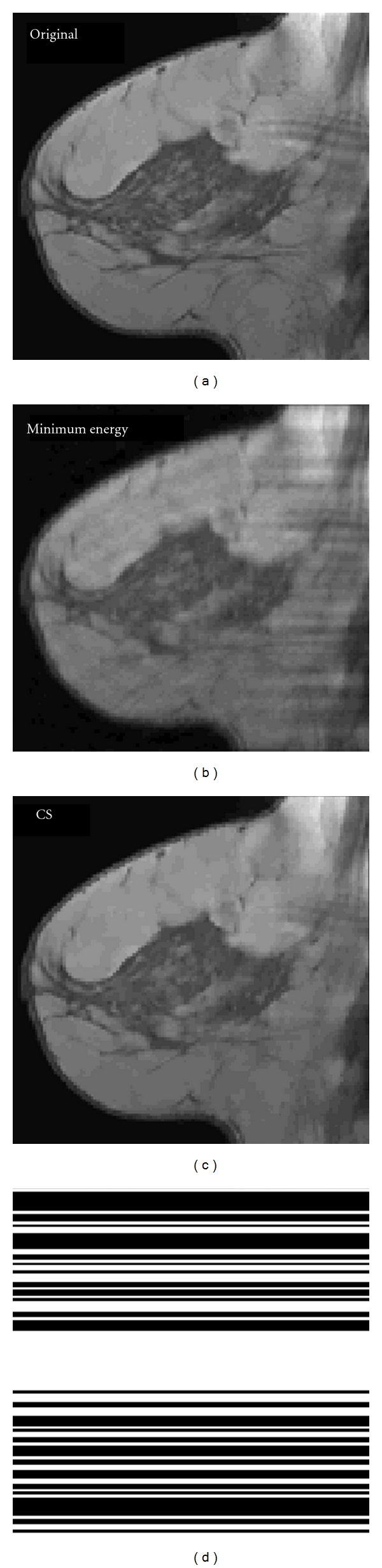
Sample reconstructions from the timing experiment. (a) shows the original *T*_1_-weighted breast image, (b) is the result of replacing the missing Fourier coefficients with zeros (minimum energy reconstruction), and (c) shows the CS reconstruction. (d) shows the 50% undersampled pattern of Fourier data retained (white is acquired; black is omitted). Entire lines of the Fourier domain were chosen to be consistent with the constraints of a 2D MRI acquisition.

 The second experiment, and the focus of this work, was a CS MRI reconstruction of a *T*_1_-weighted breast image subjected to a 50% undersampling in Fourier (spatial frequency) space. Total variation was used as the sparsity constraint and was defined as the sum of the magnitudes of pixels in the gradient image. (See [5] for more details about CS MRI reconstruction in general.) Figure 1 shows sample images from the experiment and the random Fourier sampling pattern.

CS MRI reconstructions were performed for powers-of-two image sizes ranging from 32^2^ to 8192^2^ (up to 4096^2^ only for double precision, due to memory limitations). This range covers the range of realistic MR acquisition matrix sizes for 2D scans with allowance for specialty techniques at very low or very high resolutions or future developments in imaging capabilities. The largest matrix sizes can also be indicative of the performance of three-dimensional reconstruction problems. For example, a 256^3^ data set is the same size as a 4096^2^ one.

 The CPU-based reconstructions were performed with and without multithreading enabled. All reconstructions were timed eleven times, with the first iteration discarded and the following ten iterations averaged. This avoids biasing the results with startup costs associated with both Jacket and CUDA. Jacket uses Just-in-Time (JIT) compilation to improve performance of repeated function calls, so the first call to the Jacket library is slowed by this compilation step. The CUDA driver, upon initial invocation, optimizes the low-level GPU code for the particular hardware being used. These two processes increase code performance across multiple runs but reduce it for the initial function calls.

 For timing experiments, the startup penalty is a confounding factor. Accurate timing thus requires that the reconstruction be “warmed up” with a similar problem before timing the full-scale computation. Here we used the simplest approach of discarding the first iteration. In principle, though, the warmup problem can be much smaller in data size as long as it uses the same set of Jacket functions needed in the full-size reconstruction.

## 3. Results

### 3.1. Baseline

Table 1 shows the result of the matrix multiplication experiment with and without CPU multithreading enabled. In terms of CPU performance, Matlab R2010b accelerated the CPU-based matrix multiplication by ~6 on this six-core processor using multithreading. Using the GPU then yielded an additional factor of ~5 beyond this, with a combined speedup of ~30 over a single CPU core. Based on the measured GPU single- and double-precision performance of 650 and 311 GFLOPs, respectively, we can see that Matlab R2010b combined with Jacket 1.8.0 reached 60%, and 63% of the theoretical maximum single- and double-precision performance, respectively, of the GPU.

### 3.2. CS MRI Reconstruction

**Figure 2:**
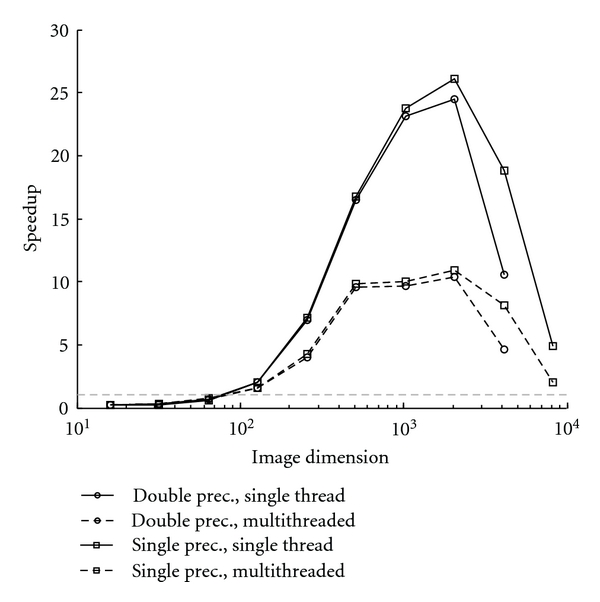
Speedup factors for the CS MRI reconstruction as a function of image size with and without CPU multithreading. The gray, dashed horizontal line shows a speedup of one.

The results of the CS MR image reconstructions are shown in Tables 2 and 3 and Figure 2. The maximum speedup was 27 for a single-precision image of size 2048^2^. For a typical double-precision MRI acquisition matrix of 256^2^ to 512^2^, we found speedups of ~7–17.

Figure 2 shows the speed advantage of the GPU-based code over using both one and multiple CPU cores. The difference between enabling multithreading or not was large, and should be considered to fairly evaluate the speed improvement of using a GPU. As can be seen in Figure 2, the speedup was less than one for images smaller than about 64^2^, followed by a rapid gain in speedup factor for images of 2048^2^ and a decline in speedup factor for the largest matrix sizes. Despite the performance falloff, the largest image was reconstructed over an order of magnitude faster on the GPU. The single-precision GPU code was able to reconstruct images up to 8192^2^ before running out of GPU memory since single-precision matrices require half the storage per element of double-precision matrices.

## 4. Discussion

Two notable features are evident in Tables 2 and 3. First, the GPU reconstruction times are very similar for images below 256^2^, regardless of numerical precision. This is due to the communication overhead in transferring the data from the CPU memory to the GPU memory. For the smallest images, this cost dominated the computation time and, for images below 64^2^, even caused the GPU reconstruction to take longer than the CPU reconstruction. This suggests that for very small acquisition matrices an efficient GPU reconstruction should combine multiple 2D data sets, such as different slices or echoes, into one reconstruction problem.

 The second interesting feature of Tables 2 and 3 is that GPU reconstruction times for images of size 1024^2^ and smaller were effectively instantaneous. A typical rapid gradient echo sequence may employ a repetition time of 5 ms, so a 50% undersampling of an *N* ^2^ Fourier matrix would require roughly 2.5 *N* ms to acquire (ignoring other acceleration methods for simplicity). The smallest image tested here would thus take 40 ms to acquire, while the CS reconstruction would take only 60 ms to complete, even with the severe communication penalty. The largest double-precision image we tested (4096^2^) would take 15 s to acquire and 10 s to reconstruct. Thus our GPU-based platform has the capacity to produce iteratively reconstructed CS-accelerated gradient echo images in real time for some MRI applications.

 The decline in acceleration at 4096^2^ was likely due to memory limitations (Yalamanchili, private communication). The NVIDIA Tesla C2050 card is designed such that each streaming multiprocessor (SM; comprised of 32 floating point units) shares a single 64 KB block of local memory. For a double-precision complex matrix multiplication, each matrix element requires 16 bytes of storage, so a maximum of 4096 elements can be stored in the SM's shared memory.

 Matlab stores arrays in column major order, so a matrix column must fit entirely into the shared memory of the SM in order to minimize memory access overhead. Thus memory access patterns will become inefficient above matrix sizes of 2048 for double-precision complex matrix multiplication. We do in fact see a leveling off of performance at 1024^2^ and a dramatic decline in performance above 2048^2^, which is consistent with this prediction.

 Many alternate methods of accelerating the CS reconstruction on a GPU platform exist, including writing custom low-level code in CUDA C or OpenCL, using free low-level libraries like CULA and CUFFT, using Matlab's built-in GPU library through the Parallel Computing Toolbox (R2010b later only), or using other free high-level Matlab-CUDA interfaces, such as GPUmat.

**Figure 3:**
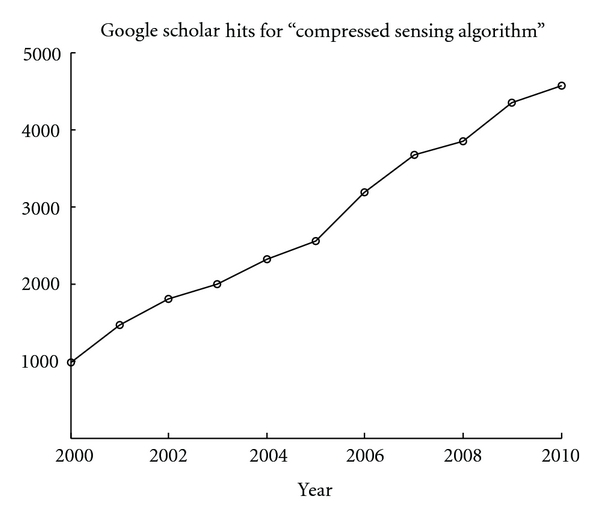
Linear growth of yearly Google Scholar hits for “compressed sensing algorithm” over the last decade. The steadily growing number indicates the increasing difficulty of keeping pace with algorithmic improvements in compressed sensing.

 The pace of algorithmic development is accelerating in compressed sensing, as demonstrated in Figure 3, and one advantage of using a high-level interface to the GPU is rapid prototyping and implementation. Debugging time is shorter with high-level languages, and coding effort is reduced, allowing the newest algorithms to be implemented quickly while still retaining a majority of the theoretical computational benefit of the GPU. Matlab's built-in GPU library does not support the array indexing needed for the gradient operation, so we could not compare it here. GPUmat is free and open source and a viable alternative to Jacket in theory, but we were unable to use it with our reconstruction code due to an unresolvable memory access error. Jacket is the only high-level software package to support sparse matrix operations on the GPU, which allows classes of compressed sensing algorithms that use sparse matrix operators to be used. (e.g., the gradient operation can be implemented as a bidiagonal matrix.) Also, the overloaded GPU functions in Jacket are implemented as MEX files, which are precompiled C/C++ functions callable from within Matlab; so writing custom CUDA subroutines could only eliminate the function call overhead and not speed up the individual SIMD operations.

## 5. Conclusion

We have shown that GPU computation significantly accelerated CS MRI reconstruction of all but the smallest of the tested image sizes. The combination of Matlab and Jacket yields a processing package that is able to realize over half of the theoretical maximum performance of the GPU, while requiring minimal code development.

 The speedup realized by the GPU for the smallest images was progressively hampered by communication overhead, while the largest images suffered from the limited GPU RAM. The optimal image dimensions, however, seem to be serendipitously close to that of high-resolution MRI data; so GPU computing, coupled with the Goldstein and Osher split Bregman algorithm, appears to be a well-suited platform for rapid CS MRI reconstruction.

 Future improvements to these methods include algorithm modifications to allow unified reconstruction of multiple two-dimensional or a single three-dimensional Fourier data set on the GPU with a single call to the reconstructor, thus reducing the communication penalty. Additional cores on the GPU card could allow higher acceleration, since we found that the split Bregman algorithm parallelizes extremely well. And finally, more RAM onboard the GPU would allow larger data sets to be reconstructed more efficiently.

## Figures and Tables

**Algorithm 1:**
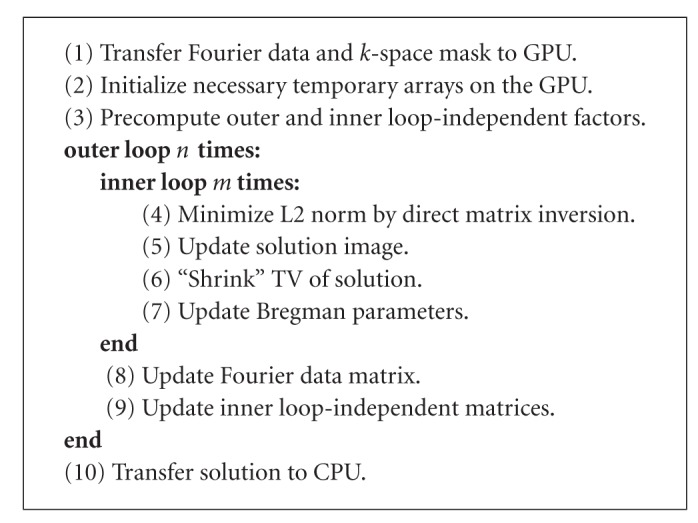
GPU-based Split Bregman Compressive Sensing Reconstruction.

**Table 1:** Measured peak CPU and GPU performance in GFLOPs for a matrix multiply experiment using Matlab and Jacket. The GPU achieves a factor of ~5 improvement over the multicore CPU.

	CPU performance (GFLOPS)		GPU performance (GFLOPS)
Precision	1 Core	6 Cores	
Single	23.6	121	650
Double	11.8	60.2	311

**Table 2:** Single-precision GPU CS MRI reconstruction times for test images ranging from 16^2^ to 8192^2^ with and without CPU multithreading. Effective speedup is the multicore CPU time relative to the GPU time.

Square	CPU time (s)		GPU	Effective
image size	1 Core	6 Cores	time (s)	speedup
16	0.011	0.011	0.060	0.18
32	0.016	0.017	0.060	0.28
64	0.038	0.046	0.063	0.73
128	0.13	0.099	0.061	1.62
256	0.49	0.28	0.066	4.24
512	2.0	1.1	0.12	9.17
1024	8.4	3.4	0.34	10.00
2048	35	14	1.3	10.77
4096	160	68	8.3	8.19
8192	670	270	140	1.93

**Table 3:** Double-precision GPU CS MRI reconstruction times for test images ranging from 16^2^ to 4096^2^ with and without CPU multithreading. Effective speedup is the multicore CPU time relative to the GPU time.

Square	CPU time (s)		GPU	Effective
image size	1 Core	6 Cores	time (s)	speedup
16	0.011	0.011	0.062	0.18
32	0.017	0.017	0.062	0.27
64	0.040	0.046	0.065	0.71
128	0.13	0.10	0.064	1.5
256	0.49	0.28	0.070	4.0
512	2.0	1.2	0.12	10
1024	8.4	3.4	0.35	10
2048	35	15	1.4	11
4096	160	69	15	5

## References

[1] Akcakaya M, Nam S, Hu P (2011). Compressed sensing with wavelet domain dependencies for coronary MRI: a retrospective study. *IEEE Transactions on Medical Imaging*.

[2] Haldar JP, Hernando D, Liang Z-P (2011). Compressed-Sensing MRI With Random Encoding. *IEEE Transactions on Medical Imaging*.

[3] Otazo R, Kim D, Axel L, Sodickson DK (2010). Combination of compressed sensing and parallel imaging for highly accelerated first-pass cardiac perfusion MRI. *Magnetic Resonance in Medicine*.

[4] Zhao B, Haldar JP, Brinegar C, Liang ZP Low rank matrix recovery for real-time cardiac MRI.

[5] Lustig M, Donoho D, Pauly JM (2007). Sparse MRI: the application of compressed sensing for rapid MR imaging. *Magnetic Resonance in Medicine*.

[6] Hansen MS, Atkinson D, Sorensen TS (2008). Cartesian SENSE and *k-t* SENSE reconstruction using commodity graphics hardware. *Magnetic Resonance in Medicine*.

[7] Sorensen TS, Schaeffter T, Noe KO, Hansen MS (2008). Accelerating the nonequispaced fast fourier transform on commodity graphics hardware. *IEEE Transactions on Medical Imaging*.

[8] Stone SS, Haldar JP, Tsao SC, Hwu WMW, Sutton BP, Liang ZP (2008). Accelerating advanced MRI reconstructions on GPUs. *Journal of Parallel and Distributed Computing*.

[9] Jia X, Lou Y, Li R, Song WY, Jiang SB (2010). GPU-based fast cone beam CT reconstruction from undersampled and noisy projection data via total variation. *Medical Physics*.

[10] Chen SS, Donoho DL, Saunders MA (1998). Atomic decomposition by basis pursuit. *The SIAM Journal on Scientific Computing*.

[11] Kim SJ, Koh K, Lustig M, Boyd S, Gorinevsky D (2007). An interior-point method for large-scale *ℓ*1-regularized least squares. *IEEE Journal on Selected Topics in Signal Processing*.

[12] Daubechies I, Defrise M, de Mol C (2004). An iterative thresholding algorithm for linear inverse problems with a sparsity constraint. *Communications on Pure and Applied Mathematics*.

[13] Hale E, Yin W, Zhang Y (2007). Fixed-point continuation for l1-minimization: methodology and convergence.

[14] van den Berg E, Friedlander MP (2008). Probing the pareto frontier for basis pursuit solutions. *The SIAM Journal on Scientific Computing*.

[15] Figueiredo MAT, Nowak RD, Wright SJ (2007). Gradient projection for sparse reconstruction: application to compressed sensing and other inverse problems. *IEEE Journal on Selected Topics in Signal Processing*.

[16] Goldstein T, Osher S (2009). The split Bregman methods for L1 regularized problems. *The SIAM Journal on Imaging Sciences*.

